# Post‐Transplant Jejunal Mass: Mesh Erosion Masquerading as Tumor—A Case Report

**DOI:** 10.1155/crit/9708591

**Published:** 2026-08-18

**Authors:** Divya Randhawa, Angelica Hernandez Alvarez, Darian L. Hoagland, Vincenzo Villani, Devin E. Eckhoff

**Affiliations:** ^1^ Division of Transplant Surgery, Department of Surgery, Beth Israel Deaconess Medical Center, Harvard Medical School, Boston, Massachusetts, USA, harvard.edu; ^2^ Department of Surgery, Beth Israel Deaconess Medical Center, Harvard Medical School, Boston, Massachusetts, USA, harvard.edu

**Keywords:** case report, jejunal mass, kidney transplantation, mesh erosion, post-transplant lymphoproliferative disorder

## Abstract

Abdominal masses in solid organ transplant recipients present a diagnostic challenge, as immunosuppression increases the risk of malignancies such as post‐transplant lymphoproliferative disorder (PTLD) and gastrointestinal stromal tumors (GISTs). These entities frequently involve the bowel and may manifest with nonspecific or vague abdominal symptoms, making differentiation between benign and malignant small bowel obstruction (SBO) critical for appropriate management and prognosis. We present the case of a kidney transplant recipient on chronic immunosuppression who developed intermittent abdominal pain and nausea, ultimately diagnosed with partial SBO. Cross‐sectional imaging revealed a jejunal mass concerning for lymphoma. However, surgical exploration demonstrated jejunal erosion by a previously placed surgical mesh with an associated fecalith, mimicking neoplastic disease. The patient underwent segmental bowel resection with mesh removal, leading to complete resolution of symptoms. This case highlights the importance of maintaining a broad differential diagnosis when evaluating intra‐abdominal masses in transplant recipients, particularly in those with a history of prior surgery and mesh placement. Awareness of this potential mimic is essential to avoid misdiagnosis and ensure timely surgical management.

## 1. Introduction

Solid organ transplant recipients carry a substantially elevated risk of malignancy, with an approximately twofold overall increase in cancer incidence compared with the general population [1]. Non‐Hodgkin lymphoma is the most common nonskin malignancy in this population, accounting for 21% of all cancers in solid organ transplant recipients [2]. Post‐transplant lymphoproliferative disorder (PTLD), a heterogeneous spectrum of lymphoid neoplasms, occurs in 1%–2% of kidney transplant recipients and frequently involves the gastrointestinal tract, presenting as an abdominal mass, obstruction, or pain [3]. Other submucosal neoplasms such as gastrointestinal stromal tumors (GISTs) may present similarly and must also be considered in the differential diagnosis [4].

Surgical mesh, widely used for hernia repair and pelvic reconstruction, is a well‐recognized but uncommon cause of delayed gastrointestinal complications [5, 6]. Migrated mesh can serve as a nidus for bezoar or fecalith formation, producing an intraluminal mass that closely mimics neoplastic disease [7]. Pelvic mesh erosion into viscera has been reported in 1.9%–4% of cases depending on the procedure, though erosion into the small bowel is exceedingly rare, even more so in kidney transplant recipients [8]. To our knowledge, this is the first reported case of a mesh migration presenting as an intestinal mass in an adult kidney transplant recipient, highlighting that not all abdominal masses in immunosuppressed transplant recipients represent malignancies. Prior surgical materials, even decades after implantation, should remain in the differential diagnosis because timely surgical exploration may be both diagnostic and therapeutic.

## 2. Case

**Figure 1:**
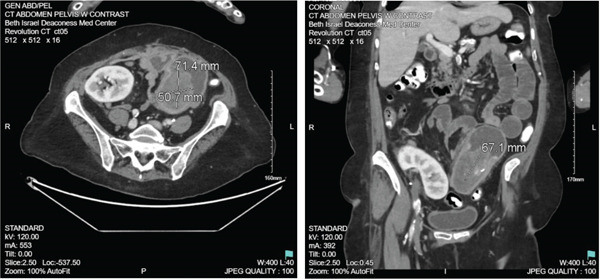
Contrast‐enhanced CT images of the abdomen showing a jejunal mass with features of partial small bowel obstruction.

**Figure 2:**
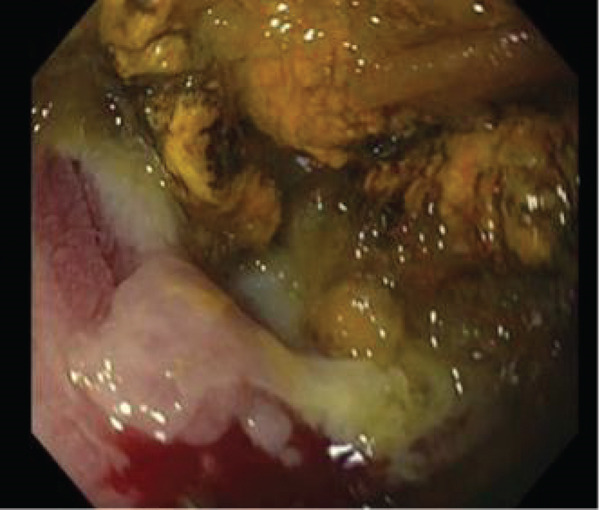
Endoscopic view revealing an intraluminal jejunal mass.

**Figure 3:**
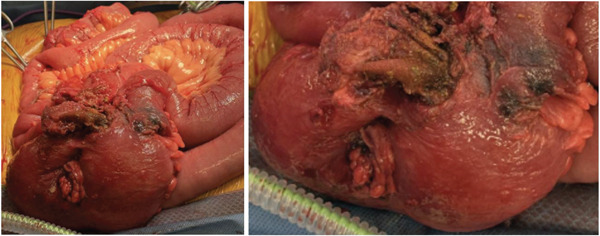
Intraoperative images showing a jejunal mass identified during exploratory laparotomy.

**Figure 4:**
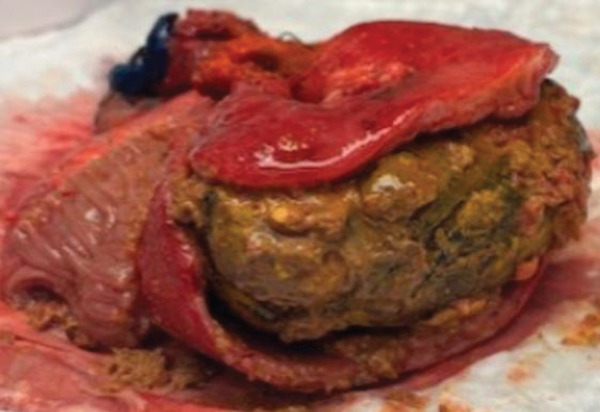
Gross pathological specimen demonstrating a fecalith within the lumen of the jejunum.

A 62‐year‐old woman with a history of right deceased donor kidney transplantation (DDKT) in 2023 for end‐stage renal disease from Type 2 diabetes presented with episodic abdominal pain, nausea, and vomiting for 3 weeks in August 2025 to Beth Israel Deaconess Medical Center. A timeline of the patient′s clinical course is provided in Table 1. Her medical history was notable for hypertension, Type 2 diabetes mellitus, and uterine cancer, for which she underwent total abdominal hysterectomy and bilateral salpingo‐oophorectomy with pelvic sling mesh placement for an unknown indication 19 years earlier. The mesh type could not be determined because operative records from the index procedure were unavailable. She denied any prior episodes of abdominal pain, bowel obstruction, or chronic gastrointestinal symptoms prior to the recent episodes. Her baseline renal allograft function was stable, with a serum creatinine of 1.2–1.3 mg**/**dL. Her original induction immunosuppression consisted of antithymocyte globulin (ATG), and she was maintained on mycophenolate sodium and tacrolimus. Contrast‐enhanced cross‐sectional imaging revealed a partially calcified intraluminal jejunal mass measuring 7.1 × 5.1 × 6.7 cm, raising concern for lymphoma or GIST in the setting of small bowel obstruction (Figure 1). Anterograde enteroscopy demonstrated a malignant‐appearing ulcerated mass in the distal jejunum with partial obstruction due to food debris proximal to the lesion (Figure 2). Endoscopic biopsies revealed fibrin, granulation tissue, and inflammation, but no evidence of malignancy. Doppler ultrasound showed no abnormalities in the transplanted kidney. Given persistent concern for malignancy and limited tissue for diagnosis, an exploratory laparotomy with small bowel resection was planned. Intraoperatively, a segment of prior mesh was found eroding into the jejunum, extending 6–10 cm intraluminally, and associated with a hard mass (Figure 3). The eroded bowel segment and associated mass were resected, and pathology revealed an 11 cm fecalith adherent to the mesh with no gross mural mass (Figure 4). Lymphadenectomy was deferred, given the intraoperative findings. The patient developed acute postoperative anemia and lactic acidosis in the recovery room and returned to the operating room for exploration and evacuation of a hematoma. The remainder of her postoperative course was uncomplicated, and she was discharged home on Postoperative Day 9. Final histopathology demonstrated small bowel with ulceration, transmural fibrosis, and serosal adhesions without evidence of malignancy. Timely surgical intervention resulted in resolution of symptoms and prevention of further mesh‐related complications such as bowel perforation. At her 2‐month surgical follow‐up, the patient reported complete resolution of abdominal symptoms, with a well‐healed incision. She tolerated a regular diet with good oral intake, and her weight remained stable during the early postoperative period, with no evidence of nutritional compromise or recurrent bowel obstruction. Maintenance immunosuppression with tacrolimus and mycophenolate sodium was continued without modification. During subsequent transplant nephrology follow‐up, tacrolimus levels remained therapeutic, no donor‐specific antibodies were detected, and renal allograft function returned to baseline (serum creatinine approximately 1.0–1.2 mg**/**dL). At her most recent follow‐up, 10 months after surgery, she remained free of recurrent gastrointestinal symptoms and continued to have stable renal allograft function.

**Table 1:** Timeline of events.

Date	Event
2006	Total abdominal hysterectomy and bilateral salpingo‐oophorectomy with a pelvic sling mesh placement
April 2023	Kidney transplant
July 2025	Initial presentation to outside hospital for 1 week of intermittent abdominal pain
August 2025	CTAP: Large necrotic mass in the distal bowel
	Anterograde enteroscopy revealed a malignant‐appearing ulcerated jejunal mass. Cold‐forceps biopsies showed granulation tissue and inflammation without evidence of malignancy
	Patient taken to the OR for exploratory laparotomy. Intraoperative findings demonstrated eroded pelvic mesh with an adherent fecalith; pathology confirmed no malignancy
	Discharged on Postoperative Day 9
October 2025	Surgical follow‐up: Complete resolution of abdominal symptoms, well‐healed incision, and stable nutritional status
March 2026	CTAP with contrast: Normal
June 2026	Transplant nephrology follow‐up: No recurrent gastrointestinal symptoms, stable renal allograft function, and continued maintenance immunosuppression

## 3. Discussion

The most significant finding of our case is that a pelvic mesh placed 19 years earlier migrated into the jejunum, formed a large fecalith, and produced an intraluminal mass that was radiologically and endoscopically indistinguishable from a post‐transplant malignancy in an immunosuppressed kidney transplant recipient. The principal diagnostic challenges were threefold: (1) the clinical and imaging features closely mimicked PTLD or GIST, entities for which the index of suspicion is appropriately high in transplant recipients; (2) standard enteroscopic biopsies failed to establish a tissue diagnosis, yielding only granulation tissue and inflammation; and (3) the absence of prior operative records precluded identification of the mesh type, limiting preoperative consideration of mesh‐related complications.

Although our patient had a negative plasma Epstein–Barr virus (EBV) viral load and no constitutional B symptoms, her presentation shared several features commonly associated with gastrointestinal PTLD, including chronic immunosuppression following kidney transplantation, an obstructing jejunal mass with mesenteric lymphadenopathy, and EBV IgG seropositivity reflecting prior EBV exposure. The overall level of immunosuppression, especially with ATG, has been strongly associated with an increased risk of PTLD [9–11]. Our patient′s regimen of ATG induction with tacrolimus and mycophenolate sodium maintenance heightened clinical suspicion for PTLD and broadened the differential diagnosis beyond more common causes of small bowel obstruction. Consequently, despite nondiagnostic biopsies, malignancy could not be confidently excluded, leading to the decision to pursue a surgical exploration.

A significant diagnostic challenge was the failure of standard enteroscopic biopsies to establish a tissue diagnosis. The American College of Gastroenterology (ACG) and American Society for Gastrointestinal Endoscopy (ASGE) guidelines state that pinch biopsies are rarely sufficient for diagnosing subepithelial lesions, achieving only a 30%–40% diagnostic yield [4, 12]. The diagnostic yield has been shown to improve with EUS guidance; however, EUS access is limited for lesions of the small bowel [13]. Although FDG PET has emerged as an important adjunct in the evaluation of suspected PTLD, it was not obtained in this patient. Cross‐sectional imaging at both the referring institution and our center consistently demonstrated a 7.1‐cm partially calcified intraluminal jejunal mass causing partial small bowel obstruction with adjacent mesenteric lymph nodes concerning for possible malignant involvement. Given the progressive obstructive symptoms and the need for definitive treatment, exploratory laparotomy was pursued as both a diagnostic and therapeutic intervention.

Mesh erosion into the bowel is a rare but well‐documented complication. A recent systematic review identified only 57 reported cases of intestinal erosion following inguinal hernia repair, with late‐presenting erosions (> 6 months postoperatively) most commonly manifesting as acute obstruction or a palpable mass [6]. Another study found that the 50th and 95th percentile time lapses between mesh implantation and excision for organ erosion were 4.6 and 17.0 years, respectively, indicating that follow‐up of more than 15 years is needed to fully assess mesh complications [14]. Our patient′s 19‐year latency falls within this extended risk window. Similar to our case, studies have described mesh migration mimicking neoplastic disease. Ojo et al. reported a migrated mesh plug presenting with features highly suggestive of an intra‐abdominal neoplasm, requiring diagnostic laparoscopy and right hemicolectomy before the mesh was identified within the specimen [5]. Chan et al. described mesh migration into the sigmoid colon 12 years after herniorrhaphy that was mistaken for a colon tumor [15]. Gallagher et al. reported a case of small bowel obstruction caused by a large bezoar firmly attached to intraluminal hernia mesh, with complete resolution of symptoms after resection—a presentation closely paralleling our case [7]. Barnes described a patient with 13 years of IBS‐like symptoms caused by Marlex mesh fully eroded into the small bowel lumen, causing partial obstruction with fecalization of enteric contents [16]. Our case is unique in that it occurred in an immunosuppressed kidney transplant recipient, where the clinical threshold to suspect malignancy is substantially lower, and the differential diagnosis is dominated by PTLD and other transplant‐associated neoplasms. The only published transplant literature on mesh‐related bowel complications involves pediatric recipients. Amir et al. reported that five of seven children who received mesh patches for abdominal wall closure after kidney transplantation developed complications, including three cases of bowel obstruction related to the surgical patch [17]. Another retrospective cohort of 146 pediatric kidney transplant recipients reported gastrointestinal surgical complications in 12 (8.2%) patients, including six cases of bowel obstruction [18]. Neither of the studies reported delayed erosion of remotely placed pelvic mesh presenting as an intraluminal mass.

Mesh migration can occur due to weakening of anatomical planes from foreign body reaction and chronic inflammation, which may be evoked by the cut edges of the mesh or improper suture placement [19]. Transplant recipients may be at heightened risk for mesh‐related complications due to the wound‐healing impairment conferred by chronic immunosuppression, which may interfere with fibroblast proliferation and collagen deposition, potentially compromising mesh incorporation and promoting migration [20]. Diabetes mellitus is an independent risk factor for mesh erosion after pelvic floor reconstructive surgery [21]. These factors could have contributed to our patient′s presentation, given her history of diabetes and immunosuppression from medications for kidney transplant.

Management of bowel erosion from mesh requires removal of the foreign material and repair or resection of the involved bowel [6]. In our case, the eroded bowel segment and associated mass were resected, and pathology confirmed an 11‐cm fecalith adherent to the mesh with no mural mass. The favorable outcome of the case presented underscores the importance of timely surgical intervention to prevent further mesh‐related complications such as bowel perforation, enteroprosthetic fistula, or complete obstruction, which have been reported in other cases of delayed mesh erosion and carry significant morbidity.

## 4. Conclusion

This case underscores that chronic immunosuppression broadens the differential diagnosis of abdominal masses but should not preclude consideration of remote surgical materials as a potential etiology. Prior surgical materials, even decades after implantation, should remain in the differential diagnosis because timely surgical exploration may be both diagnostic and therapeutic. Furthermore, nondiagnostic superficial endoscopic biopsies should not be interpreted as excluding malignancy when clinical suspicion remains high, and surgical exploration may be required for definitive diagnosis. Awareness of this rare presentation may help avoid diagnostic delay and facilitate appropriate management in transplant recipients presenting with unexplained intra‐abdominal masses.

## Funding

No funding was received for this manuscript.

## Ethics Statement

The authors have nothing to report.

## Consent

Written informed consent was obtained from the patient for the publication of this case report and any accompanying images.

## Conflicts of Interest

The authors declare no conflicts of interest.

## Data Availability

No datasets were generated or analyzed for this case report. Deidentified clinical information is available from the corresponding author upon reasonable request.
